# Fasa University Medical School: a novel experience in medical education

**Published:** 2014-01

**Authors:** HOSSAIN A. RONAGHY, KHOSROW NASR

**Affiliations:** 1 Department of Medicine University of California, San Diego, USA;; 2 Dean of Shiraz University Medical School, Shiraz, Iran

**Keywords:** Education, Medical education, Medical school

## Abstract

**Introduction: **In early 1970`s a combination of a shortage and misdistribution of health services and growing public dissatisfaction about the health care available, along with increasing expectations, has put great strain on the mind of the staff of the Department of Medicine Shiraz University School of Medicine. The purpose of this report is to give an account of what was originally planned and what has happened since the start of Fasa Medical School in April 1978.

**Methods:** This is a case report about an experience in medical education in Iran. At the time, two major problems were facing our country. The first was gross mal-distribution of these healthcare facilities, which were mostly concentrated in Tehran and big cities of Iran, and the second problem was continuous exodus of Iranian Medical graduates to the Western countries.

**Results:** The main idea of creating Fasa Medical School was to create a system in which primary care in small villages are provided by VHW with the middle level health workers of “Behdar Roustaee” to be supported by local physicians who  reside in small towns.

**Conclusion:** For Fasa Medical School, education was emphasized on community based, student centered, and problem based medical education located in the community and based on teamwork and cooperation.

## Introduction

In early 1970`s A combination of a shortage and misdistribution of health services and growing public dissatisfaction about the health care available, along with increasing expectations, has put great strain on the mind of the staff of the Department of Medicine Shiraz University School of Medicine, the medical school was known as a center of excellence for physician training. The school also got its reputation for its well-trained faculty, a fulltime system. English was the media of instruction and facilities were up to date. The Government of Iran, relatively prosperous with the sudden increase of oil price embarked on massive importation of foreign doctors, which did not, eased the aforementioned problem (1, 2).

The Fasa Medical School was conceived, to address health needs of majority of population residing in rural and small communities, by training both medical doctor and other health personnel, based on the philosophy of community orientation, problem-based learning and student-based curriculum, combined with innovations in staff and student selection and training (3).

The purpose of this report is to give an account of what was originally planned and what has happened since the start of Fasa Medical School in April 1978.

**The health manpower situation in Iran in early 70s**

In late 1960’s and early 70’s, Iran’s population was approximately 34 million and total numbers of physicians were about 14,000 a ratio of 0.41 physicians per 1000 population (4). At the time, two major problems were facing our country. The first was gross mal-distribution of these healthcare facilities, which were mostly concentrated in Tehran and big cities of Iran (1), and the second problem was continuous exodus of Iranian Medical graduates to the Western countries (5, 6).

The study that was done in 1974 indicated a permanent loss of 1624 Iranian Medical Graduates (IMG) to the United States alone (5, 6). Since over 60% of Iranian population were residing in about 50,000 small villages of 100-1000 population it was suggested that physician coverage to these scattered population would be close to impossible. Shiraz University Medical School embarked and implemented this program to cover health and sanitary measure of rural population with astonishing results (1).

The idea of Village Health Worker (VHW) was based on bare foot doctors of China, and for the first time was implemented in non- socialized market Economy Country (2).The name of VHW was “Behdar Roustaee,” and within 5 years, provided health coverage of approximately 1.5 Million of rural population of Southern Iran. Similar program was later initiated in West Azerbaijan under Auspices of Tehran University School of Public Health and WHO (7, 8). In Lorestan and Tehran also similar programs were implemented.

**Main feature of Fasa Medical School program**

The main idea of creating Fasa Medical School was to create a system in which primary care in small villages are provided by VHW with the middle level health workers of “Behdar Roustaee” to be supported by local physicians who  reside in small towns. The last step would be connecting the whole network to tertiary care system.

1. **Recruits**
  High school graduate with minimum C average from small towns of Fars province could apply. Several faculty members selecting those who would be most suitable for this program would interview applicants.  During that same period, other medical schools in Iran selected high school graduate from test results of a national examination. This usually meant the top 10% of entire nation were recruited.
2. **Faculty**
  There were 2 groups of faculty. One was the Shiraz faculty, who designed the program and wrote the case studies. The other was local faculty including 4 who were residents in community medicine and also received intensive medical education training. These 4 were the preceptors for the case studies, hospital patients and village programs. There were also physicians in the hospital, which would review and discuss patients.
3. **Facilities**
  A-Library- included 1500 books, mostly English Texts along with subscription to 10 top International Medical Journal. One US graduated information science, graduate of Brat Institute of N. Y., and 2 librarians to manage the medical library.
  B-Labs-30 Microscope with bacteriology facilities and equipment along with some pathology lab facilities with part time pathology resident from Shiraz were utilized in Fasa program.
  C-Classroom-A general conference room with capacity of 150 along with 15 different classes, equipped with projector and other audiovisual facilities were provided.
  D-Audiovisual aids- Fasa was equipped with state of art recording facilities and indeed with very close co-operation with the Dept. of Medicine and Community Medicine different professors discussed current cases, which was presented to student and were shown in group discussion.
  E-Hospital- A 50 bed red lion and sun (Equivalent to Red cross)- Hospital was already in operation and Fasa got the privilege to have student practice there later a 500 bed hospital was made adjust—to Medical School, which considered University hospital.
4. **Curriculum**
  The first 3 years out of 6, were planned and included 3 tracts:
  The first tract was case studies, one per weeks that were designed in a 3-year period to cover all major problems. Students were divided in groups of 4 or 5.They were given the patient case and had a preceptor they had one week to study, discuss, look up and be ready to discuss at the end of one week with their other classmates, who had the same case. These patient studies were designed in such a way as to permit discussion of basic science and pathophysiology of disease. The case studies started simple and became more complex. But always the emphasis was on problem solving and self-learning.
  The second tract was clinical work in the hospital. Again in groups of 4 to 5, they were given a patient. Initially they took history and similar to the case studies studied and discussed the patient with preceptor. Gradually, physical exam was added.
  The third tract was “village health.” Each4- 5 students were given a village and simply told that in 3 years, we expected improvement of that village’s health. Defining and solving the problem was left to the students… again, they had preceptors and discussion periods.
   Two classes of students, each with 18 students were selected prior to the Revolution and Cultural Revolution. Of the 36 students admitted to 2 classes 26 were transferred to Shiraz University and graduated with Shiraz classmates. (The remaining 10 were either expelled or dropped from the from the program). All 26 graduates from this program are presently (August 2013) practicing in Iran and 69% of them in Fars province. Unfortunately the curriculum of Fasa was not continued but the concept of local schools in smaller cities has been established with a larger physician output.

## Conclusion

Fasa School of Medicine was established to meet the needs of the underserved and smaller communities, for Shiraz University it emphasized the importance of relevance of medical education. For Fasa Medical School, education was emphasized on community based, student centered, and problem based medical education located in the community and based on teamwork and cooperation. Though its program was interrupted, it did show that medical students don’t have to be selected from the top grades and basic science taught in the classic method is not essential. 
